# Syndrome-specific antimicrobial escalation and diagnostic stewardship gaps in large-scale Hungarian pig farms: a cross-sectional survey

**DOI:** 10.3389/fvets.2026.1864286

**Published:** 2026-07-15

**Authors:** Ádám Kerek, Mátyás Ágics, Péter Máté, István Makkai, László Búza, Marietta Máté, László Ózsvári

**Affiliations:** 1 Department of Pharmacology and Toxicology, University of Veterinary Medicine Budapest, Budapest, Hungary; 2 National Laboratory for Infectious Animal Diseases, Antimicrobial Resistance, Veterinary Public Health and Food Chain Safety, University of Veterinary Medicine Budapest, Budapest, Hungary; 3 Department of Veterinary Forensics and Economics, University of Veterinary Medicine Budapest, Budapest, Hungary; 4 MSD Animal Health Hungary, Budapest, Hungary; 5 Department of Food Hygiene, University of Veterinary Medicine Budapest, Budapest, Hungary

**Keywords:** antimicrobial stewardship, diagnostic stewardship, group medication, one health, susceptibility-guided sampling, swine production, veterinary public health

## Abstract

**Background:**

Antimicrobial stewardship in pig production depends not only on which drugs are used, but also on how syndrome burden is recognized, when therapy escalates from individual to group level, whether bacteriological and susceptibility-guided sampling is performed, and how medicines are recorded, stored, and administered. We investigated these connected decision layers in large-scale Hungarian pig farms.

**Methods:**

Between November 2020 and March 2021, a questionnaire-based, respondent-reported cross-sectional survey was conducted among veterinarians and, where needed, farm managers or owners from 16 commercial pig farms across five Hungarian regions. Four production-unit categories were evaluated—farrowing units (*n* = 14), nursery units (*n* = 16), finisher units (*n* = 12), and breeding herds (*n* = 14), resulting in 56 production-unit observations. Syndrome burden was scored for respiratory, gastrointestinal, joint/neurological, and urogenital disease groups, together with isolation practices, treatment escalation, bacterial culture and antimicrobial susceptibility testing, and selected stewardship indicators.

**Results:**

Antimicrobial decision-making was strongly syndrome- and stage-specific. Respiratory burden was highest in nursery units (93.8%) and finisher units (91.7%), gastrointestinal burden in farrowing units (100.0%) and nursery units (87.5%), joint/neurological burden in nursery units (100.0%), and urogenital burden in breeding herds (85.7%). Group treatment was common for gastrointestinal (76.9%) and respiratory syndromes (67.5%), but rare for joint/neurological syndromes (9.3%) and absent for urogenital syndromes. Bacterial culture and antimicrobial susceptibility testing were reported in only 46.2–61.5% of affected units, depending on syndrome group. Important stewardship gaps were also identified: only 41.1% of production units used electronic records, 62.5% stored antibiotics in locked locations, 60.7% lacked post-opening expiry labeling, and 16.1% acknowledged at least some reuse of expired products.

**Conclusion:**

Large-scale pig-farm antimicrobial management was not diffuse; it followed syndrome-specific therapeutic logics. However, the diagnostic and operational infrastructure supporting prudent use remained inconsistent. The most actionable gains lie in strengthening early segregation of clinically affected animals, susceptibility-guided diagnostic support, record quality, and the procedural discipline of medicine handling.

## 1 Introduction

Antimicrobial resistance (AMR) is a One Health problem that links animal health, farm productivity, food safety, environmental dissemination, and the future effectiveness of antibacterial therapy in both veterinary and human medicine. In pig production, antimicrobials remain indispensable for preserving welfare (One Welfare) and controlling bacterial disease, yet pig farms have historically contributed substantially to veterinary antimicrobial consumption and therefore to the broader stewardship debate (1–5). The central challenge is not whether antimicrobials should ever be used in pigs, but how to ensure that use remains justified, targeted, and operationally disciplined.

This question is especially important in intensive swine systems, where disease pressure is strongly structured by production stage. Respiratory disease complexes dominate in nursery and finisher pigs and are classically linked to *Mycoplasma hyopneumoniae*, *Actinobacillus pleuropneumoniae*, *Pasteurella multocida*, and *Bordetella bronchiseptica*, with large downstream effects on daily gain, feed efficiency, mortality, and culling (6–14). Enteric syndromes are similarly production-stage dependent, spanning neonatal and post-weaning *Escherichia coli*-associated disease, swine dysentery, and proliferative enteropathy caused by *Lawsonia intracellularis*, all of which can strongly amplify demand for oral group medication when prevention is suboptimal (3, 15–20).

Other syndrome classes are no less relevant for stewardship, even if their therapeutic logic differs. Joint and neurological disease in pigs is often linked to *Glaesserella parasuis* and *Streptococcus suis*, conditions in which progression, tissue tropism, and case fatality can prompt rapid individual intervention rather than immediate whole-group medication (21–24). In breeding herds, urogenital and reproductive disorders such as leptospirosis create a different management problem, where transmission control, vaccination, rodent control, and herd-level biosecurity are at least as important as treatment itself (12, 25, 26). Consequently, prudent antimicrobial use in pigs cannot be evaluated adequately without understanding how disease-group-specific epidemiology translates into distinct treatment thresholds, sampling strategies, and practical administration routines.

For that reason, antimicrobial stewardship in livestock should be studied as an operational system rather than as a drug-consumption total alone. At farm level, stewardship is shaped by whether sick animals can be separated, whether treatment remains individual or escalates to group level, whether bacteriology and susceptibility testing are performed, who administers the medicines, how records are maintained, and whether storage and dosing practices protect against avoidable misuse (1, 27, 28). Recent work from Dutch pig systems has further shown that actual antimicrobial use is tightly linked to herd-health status and to specific disease syndromes, particularly in weaners, reinforcing the need to analyze disease occurrence and management decisions together rather than separately (29).

Despite growing attention to AMR, most farm-level pig studies still emphasize either pathogen-specific resistance, drug-class consumption metrics, or qualitative perspectives on responsible use, while fewer datasets simultaneously describe disease burden, treatment escalation, resistance-oriented diagnostics, and administration practices across production units within commercial farms (1, 2, 30–32). This gap is particularly relevant in Central and Eastern Europe, where integrated farm-level stewardship evidence remains limited in the indexed literature.

Conceptually, the present study addresses an upstream layer of AMR risk that is often under described in farm-level pig research: the operational decision architecture that determines when antimicrobial exposure becomes broader, less targeted, and less diagnostically supported. Even in the absence of isolate-level resistance phenotypes, this layer is highly relevant to AMR because it shapes the intensity, timing, and selectivity of antimicrobial pressure at production-unit level. In that sense, disease burden, treatment escalation, diagnostic support, and medicine-handling quality should be interpreted not as separate management variables, but as interconnected determinants of stewardship quality.

The aim of the present study was to characterize syndrome-specific antimicrobial management patterns in large-scale Hungarian pig farms, with particular emphasis on the interface between disease burden, therapeutic escalation, bacteriological and susceptibility-oriented diagnostic support, and practical stewardship implementation. We hypothesized that respiratory and gastrointestinal syndromes would drive the greatest reliance on group medication, whereas the supporting stewardship infrastructure—including segregation, diagnostic follow-up, recording, storage, and administration—would remain heterogeneously implemented across production units.

## 2 Materials and methods

### 2.1 Study design and farm recruitment

The study was designed as a questionnaire-based, respondent-reported cross-sectional survey of commercial Hungarian pig farms. Data collection was performed between November 2020 and March 2021. Convenience sampling was applied. Farms were contacted by e-mail, telephone, and in person. A total of 32 farms were approached, 21 agreed to participate in the broader survey campaign, and the present stewardship-focused analysis includes the 16 farms with sufficiently complete and internally consistent responses for the questionnaire domains analyzed here. Farms were eligible if they had (i) at least 500 breeding sows and their progeny or a comparably large finishing population, (ii) continuous recording of population and production data, (iii) detailed digital herd-health records including laboratory findings, morbidity, treatments, and vaccinations, and (iv) willingness to participate. Farms with incomplete or inconsistent veterinary health records were not retained in the final analytical dataset. In the present study, the term “large-scale” was used as an operational study category defined by these restrictive inclusion criteria rather than by a formal legal threshold alone. Sixteen large-scale farms from five Hungarian regions were included: four farms each from the Northern Great Plain, Southern Transdanubia, and Central Transdanubia, three from Western Transdanubia, and one from the Southern Great Plain. Seven farms operated as breeding-to-finishing farms, seven as breeding-only farms, and two as finishing-only farms. The survey was completed primarily with the responsible veterinarians; in some farms, managers or owners also contributed additional operational information. Each survey session lasted approximately 30–60 min and was completed individually. To reduce interpretation bias, questionnaires were completed interactively either during on-farm visits or, when epidemiological constraints required, by video call. No formal cross-checking against veterinary prescriptions, farm purchase records, or medicine-use logs was performed; therefore, the dataset should be interpreted as respondent-reported stewardship information. Industry-affiliated co-authors contributed to farm contact and, in selected cases, to the practical organization of questionnaire collection, but they did not determine eligibility criteria, influence sample composition, or control data interpretation.

In total, 31,026 breeding sows were kept in the surveyed herds, which, according to the data published by the Hungarian Central Statistical Office (HCSO) on 1 December 2020 (33), represented almost 20% of the Hungarian sow population. Farm-level production-unit coverage and the respondent framework retained in the archived source materials are summarized in Supplementary Table S1.

### 2.2 Questionnaire structure and analytical unit

Two study-specific, harmonized questionnaires were used. The instruments were developed collaboratively by university-based researchers and swine-health practitioners, and a pilot version was tested before final use. The same swine-health specialist veterinarian administered the final survey across all farms. Responses were recorded on paper forms during the survey session and subsequently entered into Excel under GDPR-compliant data handling. No formally validated AMU questionnaire was adopted verbatim; instead, the instruments were designed as descriptive stewardship-mapping tools tailored to large commercial Hungarian pig production. The full questionnaire is provided as Supplementary File S1. The first questionnaire captured general farm descriptors, production indicators, and health-status variables. The second focused on bacterial disease occurrence, isolation practices, treatment strategy, bacterial culture and antimicrobial susceptibility testing, antimicrobial record-keeping, storage, and administration practices. For the present manuscript, the main analytical unit was the production unit. The analysis was performed at production-unit level and included farrowing units, nursery units, finisher units, and breeding herds, together comprising 56 production-unit observations. Separate grower units were present in only three farms and were not analyzed separately in the main manuscript in order to avoid unstable stage-specific estimates.

### 2.3 Variables of interest

For each production unit, respondents scored the respondent-perceived burden of four syndrome groups—respiratory, gastrointestinal, joint/neurological, and urogenital—using an ordinal scale with the categories absent, low, moderate, high, and very high. Scoring referred to the second half of 2020 and reflected an integrated field judgement of routine clinical occurrence, within-unit spread or persistence, clinical severity, and practical management relevance within the production unit, rather than a fixed numeric prevalence threshold. Operationally, absent indicated no meaningful syndrome occurrence; low indicated sporadic or limited occurrence without clear group-level impact; moderate indicated clearly recurring but operationally manageable burden; high indicated frequent and clinically important burden with evident treatment relevance; and very high indicated widespread or repeatedly disruptive burden with major clinical and operational impact. These operational definitions are summarized in Supplementary Table S2. For units in which a given syndrome was reported, additional questions addressed whether affected animals were isolated, whether treatment was individual only, immediate whole-group treatment, or threshold-based escalation to group treatment, if threshold-based escalation was used, the approximate clinical involvement threshold for switching to group medication, and whether bacterial culture and antimicrobial susceptibility testing were performed to guide treatment decisions. The full distribution of threshold-based escalation triggers by syndrome group and production stage is provided in Supplementary Table S3. Separate items addressed antimicrobial record-keeping, storage security, post-opening expiry labeling, use of expired products, the availability of water-medication technology, and selected injection-related practices. Questionnaire-derived variables included in the main analysis, together with questionnaire item mapping and stage-specific analytical denominators, are detailed in Supplementary Table S4. For consistency, respondents were asked to base their answers on the second half of 2020. No formal external verification of responses was performed beyond interactive questionnaire completion and post-entry internal consistency checks.

For analytical purposes, affected units were defined as production units with any non-zero syndrome burden, that is, units scored as low, moderate, high, or very high. Percentages for isolation, treatment strategy, and bacterial culture and antimicrobial susceptibility testing were calculated using syndrome-specific affected-unit denominators.

### 2.4 Statistical analysis

The primary analytical objective was descriptive. Categorical data are presented as counts and percentages. Disease-burden distributions were summarized by syndrome group and production stage, while management responses—animal isolation, treatment strategy, and bacterial culture and antimicrobial susceptibility testing—were summarized among affected units only. To support between-syndrome comparisons, proportions were additionally compared using exact tests, and exact binomial 95% confidence intervals were calculated for key proportions. Because multiple production units originated from the same farm, the inferential results were interpreted cautiously and primarily as descriptive statistical support. All analyses were performed in R version 4.5.3.

When item-level non-response occurred, percentages were calculated using the available denominator for that variable. Missing responses were handled by complete-case summarization at the variable level, and no imputation was performed. The corresponding denominator is shown explicitly in the relevant table or figure legend where needed. Stage-specific analytical denominators for all questionnaire-derived variables used in the main manuscript are summarized in Supplementary Table S4, and a variable-level summary of available and missing observations is provided in Supplementary Table S5. Exact syndrome-specific proportions, exact 95% confidence intervals, and omnibus exact test results for the main management-response comparisons are additionally summarized in Supplementary Table S6.

Because multiple production units originated from the same farm, the analytical dataset had a clustered structure. This was taken into account in the interpretation of the results but not modelled explicitly in a multilevel framework; therefore, exact *p*-values should be interpreted cautiously and primarily as descriptive support for between-syndrome contrasts rather than as population-level inferential estimates. A STROBE checklist is provided as Supplementary File S2.

### 2.5 Ethical considerations

Written informed consent was obtained from all participants before survey administration. Responses were recorded on paper forms during the survey session and subsequently entered into Excel in pseudonymized form under GDPR-compliant data handling. No audio recordings were used. The study did not involve experimental animal procedures, animal interventions, biological sampling, or the collection of personal sensitive data; it relied exclusively on farm-management information provided by veterinarians, farm managers, and owners. Farm-level operational data were analysed and reported in aggregated form.

## 3 Results

### 3.1 Farm characteristics

The survey covered 16 commercial pig farms and 56 analyzable production units. The farms represented both vertically integrated and specialized production structures, with a strong emphasis on breeding-only and breeding-to-finishing farms. At the herd level, the sow inventory among farms with breeding herds had a mean of 2,216 animals and a median of 1,617, while nursery and finisher inventories were similarly large, confirming that the dataset reflected genuinely large-scale production conditions (Table 1).

**Table 1:** Surveyed farm and production-unit characteristics.

Variable	Value
Farms surveyed	16
Survey period	November 2020 to March 2021
Regions represented	5
Breeding-to-finishing farms	7
Breeding-only farms	7
Finishing-only farms	2
Farrowing units analyzed	14
Nursery units analyzed	16
Finisher units analyzed	12
Breeding herds analyzed	14
Mean sow inventory (farms with breeding herds)	2,216
Median sow inventory (farms with breeding herds)	1,617
Mean nursery inventory	5,673
Mean finisher inventory	7,684

### 3.2 Respondent-reported disease burden was strongly production-stage-specific

**Figure 1:**
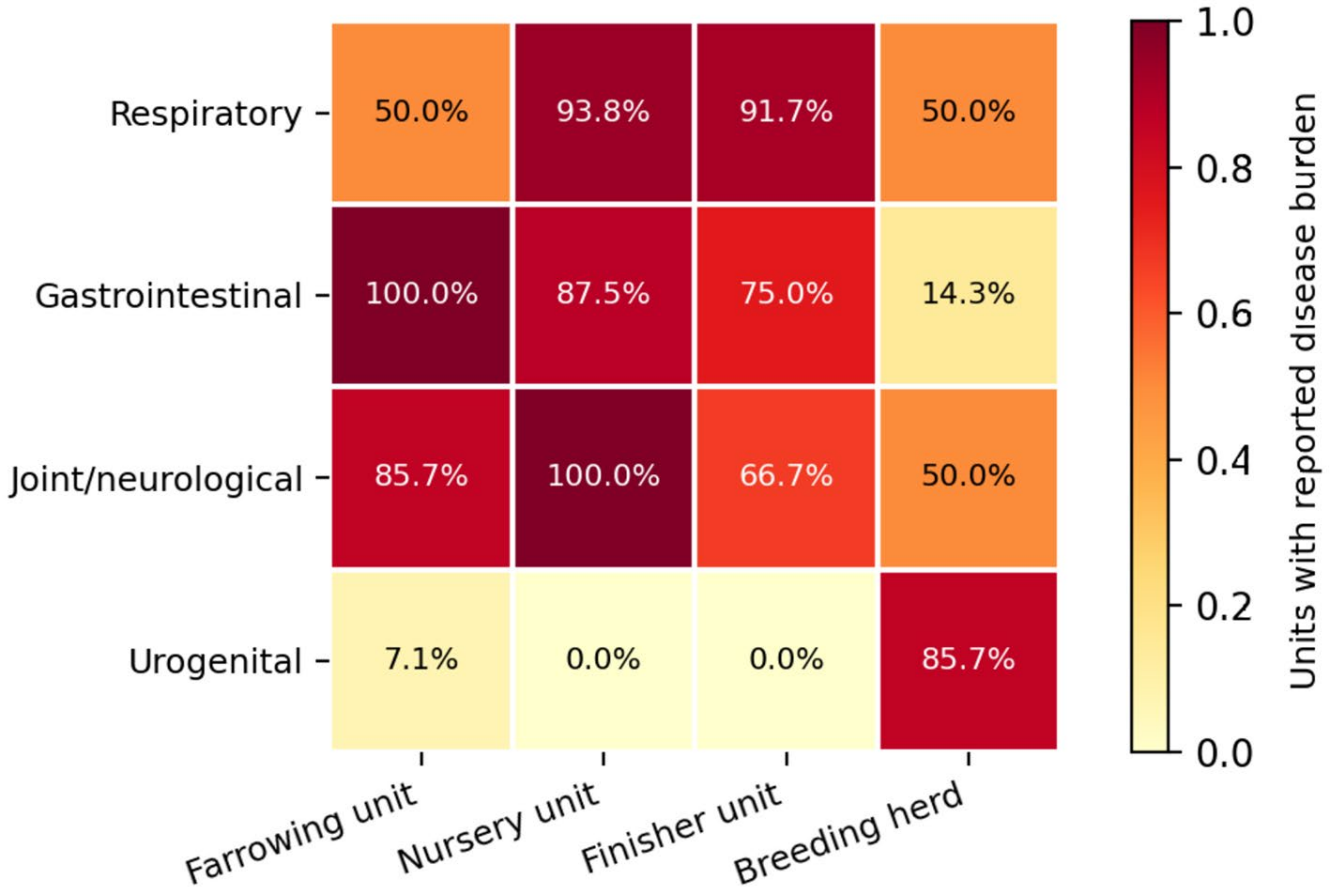
Heatmap of syndrome burden across production stages. The figure shows the proportion of production units with non-zero reported burden for respiratory, gastrointestinal, joint/neurological, and urogenital syndromes across farrowing units, nursery units, finisher units, and breeding herds. Non-zero burden includes units classified as low, moderate, high, or very high.

**Figure 2:**
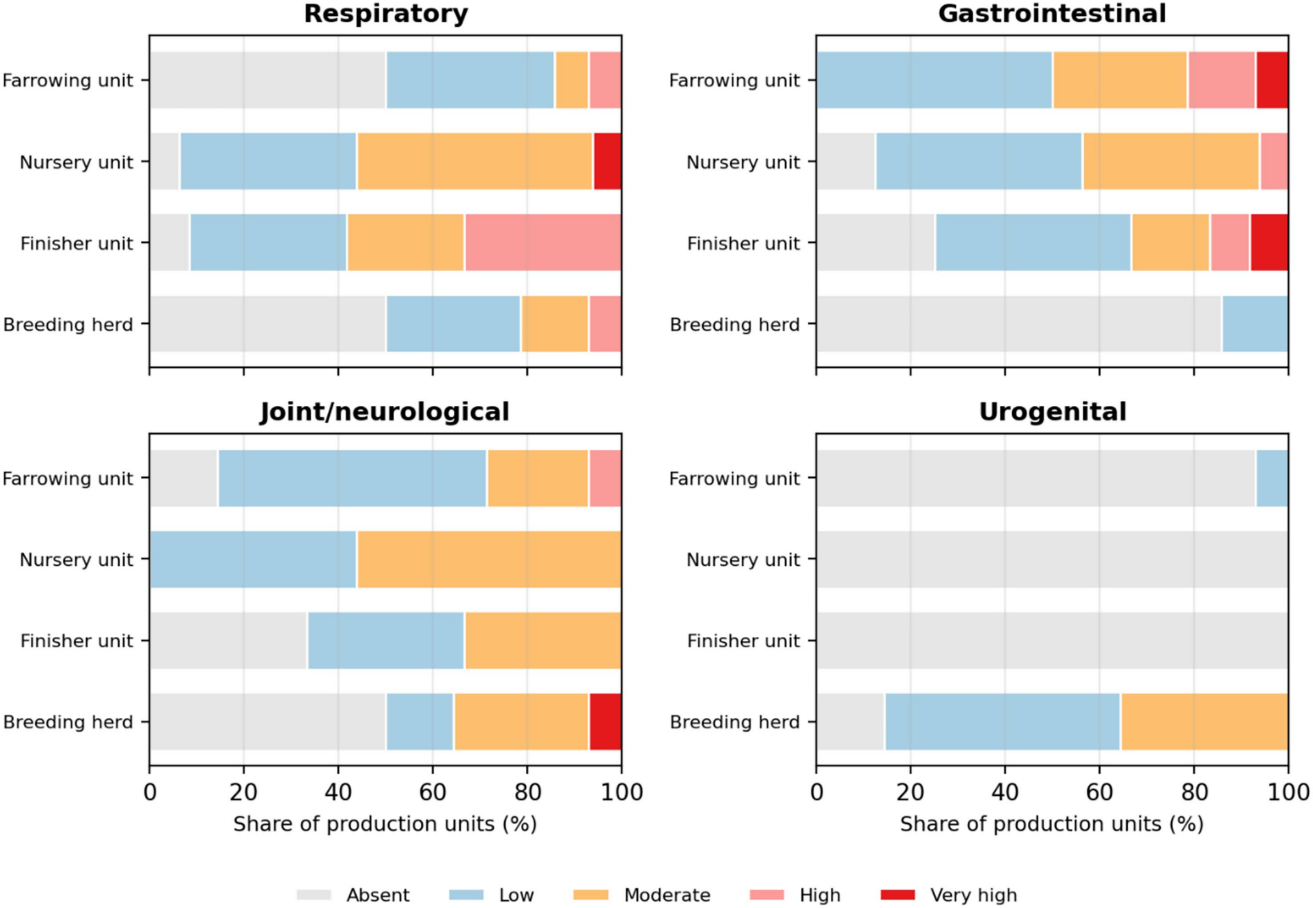
Severity distribution of reported syndrome burden by production stage. Stacked distributions show the relative frequency of absent, low, moderate, high, and very high burden categories for each syndrome group across the four production stages.

Marked stage specificity emerged in respondent-reported burden across the four syndrome groups (Figures 1, 2; Table 2). Across all 56 production-unit observations, non-zero burden was reported in 40/56 units (71.4%) for respiratory syndromes, 39/56 units (69.6%) for gastrointestinal syndromes, 43/56 units (76.8%) for joint/neurological syndromes, and 13/56 units (23.2%) for urogenital syndromes. Respiratory burden was reported in 15/16 nursery units (93.8%) and 11/12 finisher units (91.7%), but in only 7/14 farrowing units (50.0%) and 7/14 breeding herds (50.0%). Gastrointestinal burden was present in all farrowing units (14/14; 100.0%), in 14/16 nursery units (87.5%), and in 9/12 finisher units (75.0%), but in only 2/14 breeding herds (14.3%). Joint/neurological burden was likewise common in nursery units (16/16; 100.0%), frequent in farrowing units (12/14; 85.7%) and finisher units (8/12; 66.7%), and less common in breeding herds (7/14; 50.0%). In contrast, urogenital burden was concentrated in breeding herds (12/14; 85.7%) and was nearly absent from the other production stages.

**Table 2:** Presence of syndrome burden by disease group and production stage.

Production stage	Disease group	Units with reported burden
Farrowing unit	Respiratory	7/14 (50.0%)
Farrowing unit	Gastrointestinal	14/14 (100.0%)
Farrowing unit	Joint/neurological	12/14 (85.7%)
Farrowing unit	Urogenital	1/14 (7.1%)
Nursery unit	Respiratory	15/16 (93.8%)
Nursery unit	Gastrointestinal	14/16 (87.5%)
Nursery unit	Joint/neurological	16/16 (100.0%)
Nursery unit	Urogenital	0/16 (0.0%)
Finisher unit	Respiratory	11/12 (91.7%)
Finisher unit	Gastrointestinal	9/12 (75.0%)
Finisher unit	Joint/neurological	8/12 (66.7%)
Finisher unit	Urogenital	0/12 (0.0%)
Breeding herd	Respiratory	7/14 (50.0%)
Breeding herd	Gastrointestinal	2/14 (14.3%)
Breeding herd	Joint/neurological	7/14 (50.0%)
Breeding herd	Urogenital	12/14 (85.7%)

### 3.3 Reported therapeutic escalation differed sharply by syndrome class

**Figure 3:**
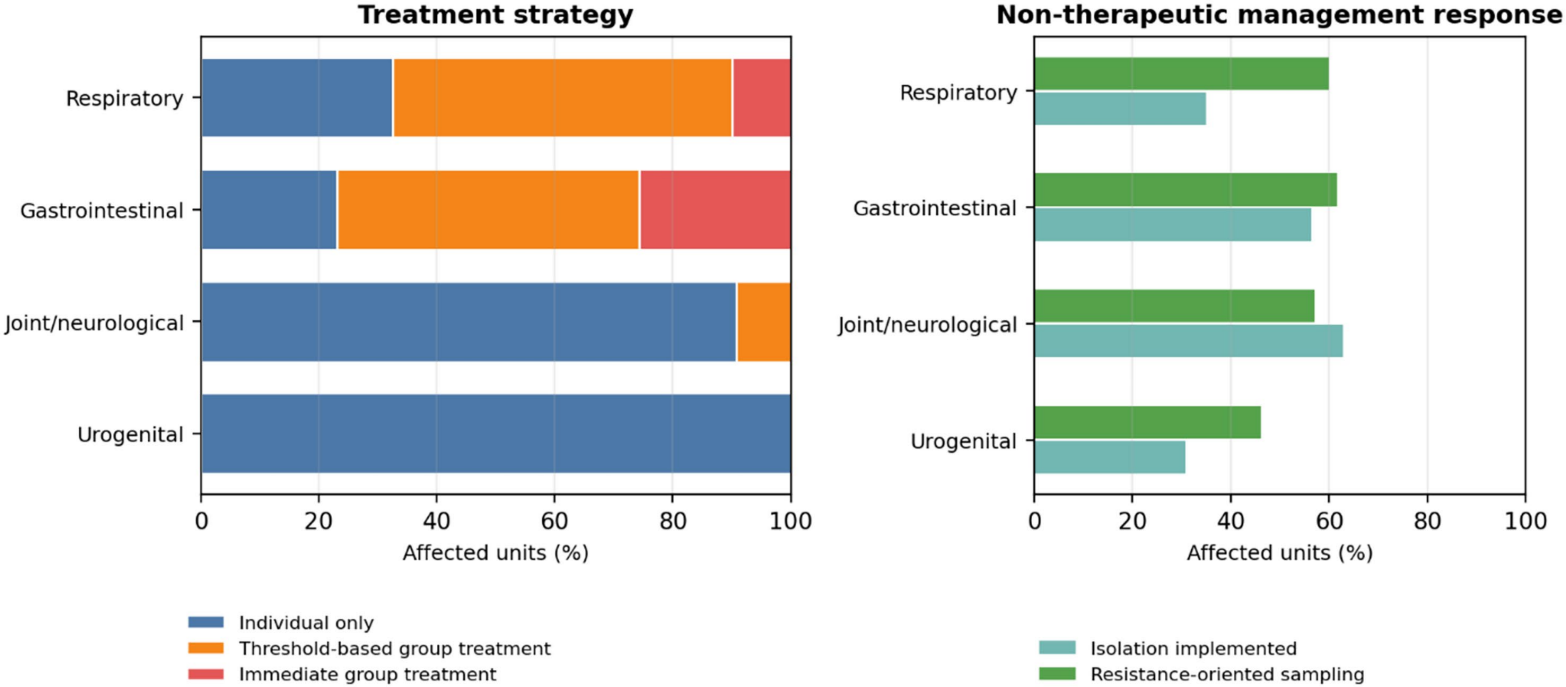
Syndrome-specific management responses among affected production units. The left panel summarizes treatment strategy among affected units as individual-only treatment, threshold-based escalation to group treatment, or immediate whole-group treatment. The right panel shows the proportion of affected units reporting animal isolation and bacterial culture and antimicrobial susceptibility testing. Percentages were calculated using syndrome-specific affected-unit denominators.

Among affected units, reported treatment strategy was not diffuse; it was syndrome-specific (Figure 3; Table 3). Group treatment, whether immediate or threshold-based, was common for gastrointestinal syndromes (30/39 affected units; 76.9%) and respiratory syndromes (27/40 affected units; 67.5%). In contrast, joint/neurological syndromes were managed almost exclusively by individual treatment (39/43; 90.7%), and all affected urogenital units were treated individually (13/13; 100.0%). Threshold-based escalation dominated over immediate whole-group treatment for respiratory syndromes (57.5% vs. 10.0%) and remained the most frequent group-level strategy for gastrointestinal syndromes as well (51.3% vs. 25.6%). Where threshold-based escalation was used, the most commonly reported trigger was clinical involvement of 11–20% of the group (Supplementary Table S3).

**Table 3:** Management response among affected production units.

Disease group	Affected units (*n*)	Isolation implemented	Individual only	Threshold-based group treatment	Immediate group treatment	Culture and susceptibility testing
Respiratory	40	14/40 (35.0%)	13/40 (32.5%)	23/40 (57.5%)	4/40 (10.0%)	24/40 (60.0%)
Gastrointestinal	39	22/39 (56.4%)	9/39 (23.1%)	20/39 (51.3%)	10/39 (25.6%)	24/39 (61.5%)
Joint/neurological	43	27/43 (62.8%)	39/43 (90.7%)	4/43 (9.3%)	0/43 (0.0%)	24/43 (55.8%)
Urogenital	13	4/13 (30.8%)	13/13 (100.0%)	0/13 (0.0%)	0/13 (0.0%)	6/13 (46.2%)

An omnibus Fisher–Freeman–Halton exact test confirmed that the frequency of group treatment differed across syndrome classes (*p* = 0.0001). Group treatment was reported in 67.5% of affected respiratory units (27/40; exact 95% CI, 50.9–81.4), 76.9% of affected gastrointestinal units (30/39; 95% CI, 60.7–88.9), 9.3% of affected joint/neurological units (4/43; 95% CI, 2.6–22.1), and 0.0% of affected urogenital units (0/13; 95% CI, 0.0–24.7). In pairwise Fisher exact comparisons, group treatment was more frequent for respiratory than for joint/neurological syndromes (*p* < 0.001) and urogenital syndromes (*p* < 0.001), and more frequent for gastrointestinal than for joint/neurological syndromes (*p* < 0.001) and urogenital syndromes (*p* < 0.001), whereas respiratory and gastrointestinal syndromes did not differ materially (*p* = 0.453).

### 3.4 Reported isolation and culture-and-susceptibility testing were unevenly implemented

Management responses other than treatment were also heterogeneous (Figure 3; Table 3). Isolation of affected animals was reported in only 14/40 respiratory syndrome-affected units (35.0%), compared with 22/39 gastrointestinal syndrome-affected units (56.4%) and 27/43 joint or neurological syndrome-affected units (62.8%). Urogenital syndromes showed the lowest absolute isolation frequency after respiratory disease (4/13; 30.8%). Reported bacterial culture and antimicrobial susceptibility testing were more consistent than isolation, but still far from universal: sampling was reported in 24/40 respiratory syndrome-affected units (60.0%), 24/39 gastrointestinal syndrome-affected units (61.5%), 24/43 joint/neurological syndrome-affected units (55.8%), and 6/13 urogenital syndrome-affected units (46.2%). Thus, a substantial proportion of clinically affected units—particularly those affected by respiratory, gastrointestinal, and urogenital syndromes—were managed without reported culture and susceptibility testing at the time captured by the survey.

An omnibus Fisher–Freeman–Halton exact test also indicated heterogeneity in isolation frequency across syndrome classes (*p* = 0.0313). Isolation was reported in 35.0% of affected respiratory production units (14/40; exact 95% CI, 20.6–51.7), 56.4% of affected gastrointestinal production units (22/39; 95% CI, 39.6–72.2), 62.8% of affected joint/neurological production units (27/43; 95% CI, 46.7–77.0), and 30.8% of affected urogenital production units (4/13; 95% CI, 9.1–61.4). Pairwise Fisher exact comparisons showed that isolation was less frequent for respiratory than for joint/neurological syndromes (*p* = 0.016), whereas the remaining pairwise contrasts were not significant (all *p* ≥ 0.058). In contrast, reported bacterial culture and antimicrobial susceptibility testing frequencies did not differ significantly across syndrome classes (omnibus exact *p* = 0.7816): 60.0% for respiratory production units (24/40; exact 95% CI, 43.3–75.1), 61.5% for gastrointestinal production units (24/39; 95% CI, 44.6–76.6), 55.8% for joint/neurological production units (24/43; 95% CI, 39.9–70.9), and 46.2% for urogenital production units (6/13; 95% CI, 19.2–74.9).

### 3.5 Record-keeping, storage and administration practices revealed stewardship gaps

**Figure 4:**
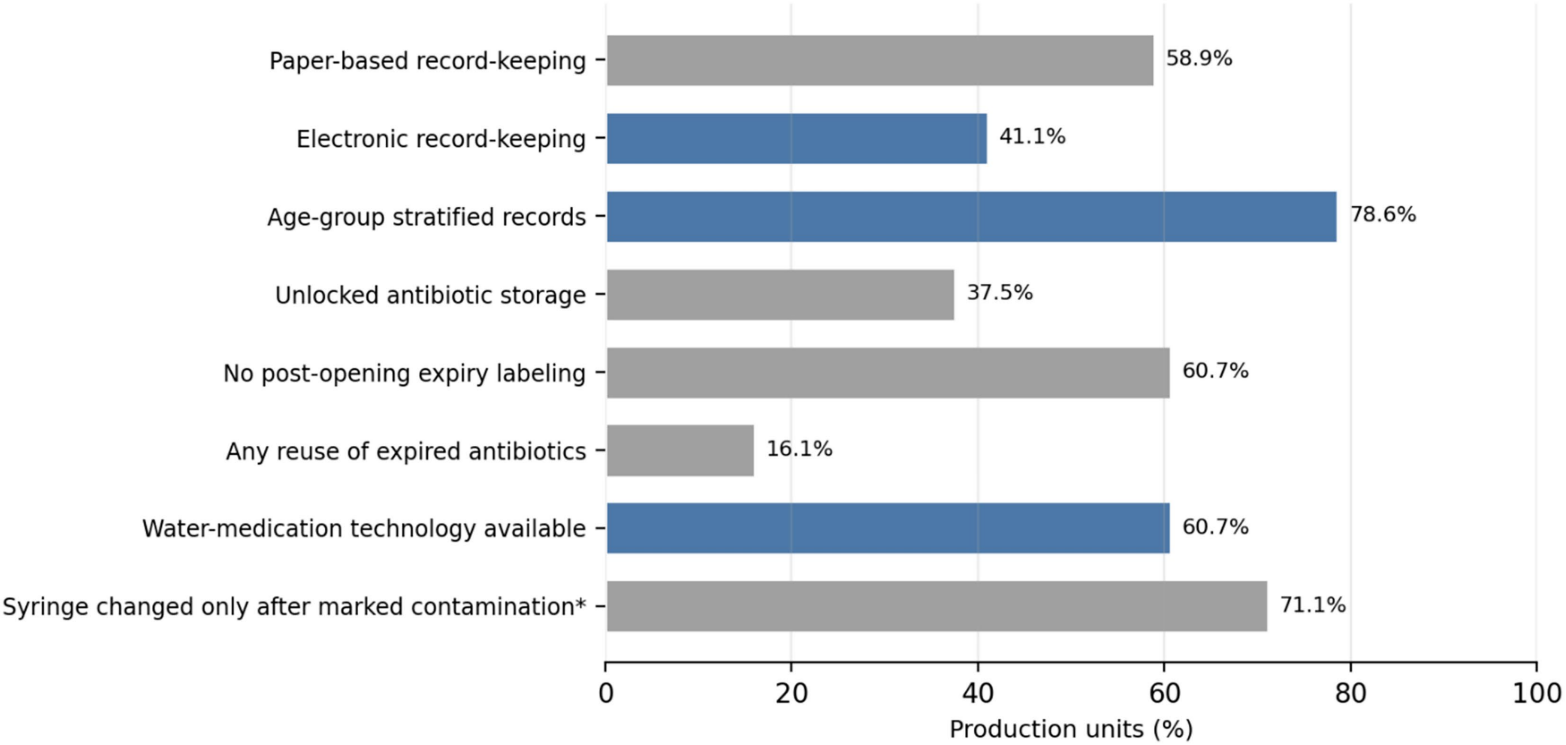
Selected antimicrobial stewardship indicators across production units. The figure summarizes key operational stewardship indicators, including record-keeping format, storage security, expiry labeling, reuse of expired products, water-medication infrastructure, and syringe-handling practices. For syringe handling, the percentage was calculated among units with an available response.

Selected stewardship indicators showed substantial variability across production units (Figure 4; Table 4). Only 23/56 production units (41.1%) used electronic record-keeping, whereas 33/56 production units (58.9%) still relied on paper-based records. Nevertheless, 44/56 production units (78.6%) reported record systems stratified by age group. Antibiotics were stored in locked locations in 35/56 production units (62.5%), leaving 21/56 production units (37.5%) without locked storage. Post-opening expiry labeling was absent in 34/56 production units (60.7%), while only 16/56 production units (28.6%) reported consistent labeling. At least some reuse of expired antibiotics was acknowledged in 9/56 production units (16.1%). Water-medication technology was available in 34/56 production units (60.7%). Among production units with available responses on syringe handling, 32/45 (71.1%) reported changing syringes only after marked contamination, indicating that practical administration routines often remained driven by convenience rather than strict preventive hygiene.

**Table 4:** Selected stewardship indicators across production units.

Indicator	Value
Electronic record-keeping	23/56 (41.1%)
Age-group-stratified records	44/56 (78.6%)
Antibiotics stored in locked locations	35/56 (62.5%)
No post-opening expiry labeling	34/56 (60.7%)
Any reuse of expired antibiotics	9/56 (16.1%)
Water-medication technology available	34/56 (60.7%)
Syringes changed only after marked contamination	32/45 (71.1%)
Needles changed only when broken	6/56 (10.7%)

## 4 Discussion

The central finding of this study is that antimicrobial management in large-scale Hungarian pig farms was structured by syndrome class rather than implemented as a diffuse or operationally uniform response to bacterial disease. Respiratory and gastrointestinal syndromes were the main triggers of group treatment, whereas joint/neurological and urogenital syndromes remained predominantly individual-treatment problems. This distinction is epidemiologically and stewardship-wise important, because it indicates that antimicrobial exposure is shaped upstream by syndrome ecology, perceived transmission dynamics, and expected production loss, rather than by drug availability alone.

This interpretation aligns closely with recent Dutch evidence showing that respiratory disease and central nervous system-related disease in weaners are among the main farm-level drivers of antimicrobial use and of group treatments in pig production (29). Our survey extends that logic by showing the decision architecture behind those treatments. In our material, respiratory and gastrointestinal syndromes did not merely occur frequently; they crossed the operational threshold beyond which farmers and veterinarians were willing to treat groups, often at relatively low reported involvement levels. This is consistent with the biological reality that both syndrome classes can spread rapidly within pens and can translate quickly into performance losses if left uncontrolled (3, 6, 11, 19, 20).

Furthermore, the survey identified a significant gap in non-therapeutic control: isolation practices were least consistent in units affected by respiratory disease, despite the fact that respiratory syndromes were among the main drivers of group treatment. The relatively low isolation frequency in respiratory disease-affected units suggests that treatment often compensates for structural or logistical limitations in within-farm segregation capacity. This interpretation is compatible with the broader literature linking internal biosecurity, all-in/all-out management, cleaning discipline, and dedicated sick pens to lower antimicrobial demand in food-animal systems (28, 29, 34). In practical terms, the most stewardship-relevant intervention may therefore not be a new drug rule, but better physical capacity for early separation of clinically affected animals.

Reported bacterial culture and antimicrobial susceptibility testing were more frequent than isolation, but they still remained inconsistent. Roughly 40% of production units with respiratory or gastrointestinal syndrome burden and almost half of production units affected by urogenital syndromes were managed without reported susceptibility-oriented sampling. For a field veterinarian, this gap is understandable: clinical deterioration, labor constraints, sampling costs, and turnaround time all act against systematic bacteriology in fast-moving herd situations. Yet recent work also shows that meaningful farm-level surveillance becomes far more informative when antimicrobial-use data and resistance monitoring are linked rather than collected in isolation. A recent study from the Midwestern United States demonstrated both the feasibility and the practical burden of combining farm-level purchase data with on-farm resistance monitoring in pig enterprises (31). Our results suggest that Hungarian large-scale systems would benefit from the same principle, even if implemented in a simpler, syndrome-triggered form.

Importantly, although the present study does not report isolate-level resistance phenotypes, it captures upstream decision nodes that are mechanistically relevant to AMR selection pressure. Every shift from isolated individual treatment to low-threshold group medication, every missed opportunity for susceptibility-guided sampling, and every lapse in documentation, storage, or administration quality may broaden antimicrobial exposure and reduce targeting precision. From a One Health and One Welfare perspective, these operational features are therefore not peripheral management details; they are part of the ecological context in which resistance can be selected, maintained, and operationally normalized.

The stewardship implications of the storage and administration findings are equally important. Electronic recording was not yet dominant, post-opening expiry labeling was frequently absent, a non-trivial minority acknowledged reuse of expired products, and syringe handling often followed a contamination-driven rather than prevention-driven logic. These are not minor technicalities. They are the hidden operating system of antimicrobial stewardship, because poor documentation, inadequate storage control, and relaxed administration hygiene all increase the probability of avoidable misuse, dosing uncertainty, or iatrogenic spread. Similar behavioral and operational dimensions have been emphasized in mixed-method and interdisciplinary livestock AMU studies, which argue that antimicrobial reduction cannot be sustained by prescriber intent alone; it requires changes in routines, incentives, infrastructure, and on-farm culture (1, 27, 35, 36).

Another notable feature of the dataset is the centrality of oral medication infrastructure. Water-medication technology was available in more than 60% of units, which is epidemiologically unsurprising in production systems where respiratory and gastrointestinal syndromes frequently trigger group-level intervention. This observation is consistent with the broader European pig-production context, in which oral antimicrobial administration remains operationally important in many systems. Although our survey did not quantify class-specific use, it clearly identifies the farm-level infrastructure that makes group medication logistically feasible and therefore stewardship-relevant (37).

These findings have a broader strategic implication. If the aim is to reduce antimicrobial dependence without compromising animal health and welfare, the most effective leverage points are likely to be preventive and managerial rather than purely restrictive. Recent policy and review literature consistently emphasizes that lower antimicrobial use in livestock is best achieved by combining better husbandry, improved housing conditions, stronger biosecurity, vaccination, monitoring, and economically realistic transition pathways rather than by relying on prohibition alone (30, 34, 38−41). The present dataset points to the same conclusion. In these Hungarian swine farms, the next gains in stewardship are likely to come from preventing the syndromes that currently trigger group treatment and from standardizing the operational quality of what happens before, during, and after antibiotics are administered.

Several limitations define the interpretive boundary of this study and should be stated explicitly. First, the dataset reflects structured expert-reported farm information rather than prospectively measured antimicrobial consumption data, so it cannot estimate AMU in mg/PCU, DDDA, or other quantitative indicators. Second, the study relied on respondent-reported questionnaire data rather than independently verified antimicrobial-use records. Accordingly, the findings may have been influenced by recall bias and social desirability bias, particularly for variables related to treatment escalation, diagnostic sampling, record-keeping, storage discipline, and administration practices. Although this does not diminish the practical value of the survey, it does define an important interpretive boundary, because reported stewardship practices may not always correspond fully to day-to-day operational reality. In addition, the structured survey instrument necessarily captured contextual information in simplified form and did not reconstruct full clinical histories, previous laboratory findings, cumulative farm history, or the complete veterinary reasoning that may have informed treatment decisions at the time. These constraints should be considered when interpreting the diagnostic-stewardship implications of the results. Third, the sample size, although substantial for an interview-based multi-region survey, remains modest for causal inference and is not intended to represent all Hungarian pig farms statistically. Fourth, because the data were collected in 2020–2021, the manuscript should be interpreted as a farm-management and stewardship profile of that period rather than as a direct estimate of current national practice. Another limitation is that the 56 production-unit observations were not fully independent, because multiple production units originated from the same farm and may have shared infrastructure, management culture, veterinary oversight, and stewardship routines. As a result, the exact tests used in the study may overstate statistical separation to some extent, and the inferential results should therefore be interpreted cautiously.

These limitations are balanced by several strengths. The study integrates disease burden, therapeutic decision-making, diagnostic behavior, and practical medicine handling within the same farm-level framework. It does so at production-unit resolution, which is exactly where stewardship decisions are actually made. That level of operational detail is often lost in national sales statistics and is difficult to reconstruct from laboratory surveillance alone. Accordingly, the present survey should not be viewed as a substitute for quantitative AMU or isolate-level AMR surveillance, but as a complementary layer that reveals the management logic from which those downstream signals emerge.

## 5 Conclusion

Large-scale Hungarian pig farms showed clear syndrome-specific antimicrobial management patterns. Respiratory and gastrointestinal syndromes were the dominant triggers of group treatment, whereas joint/neurological and urogenital syndromes were managed primarily at individual level. Bacterial culture and antimicrobial susceptibility testing were present but not routine, and multiple stewardship gaps persisted in record-keeping, storage, labeling, and medicine administration. The most realistic path toward lower antimicrobial dependence in such systems is therefore not indiscriminate restriction, but a more disciplined stewardship architecture built on syndrome-specific prevention, earlier segregation of clinically affected animals, more consistent bacteriological and susceptibility-guided support, and tighter procedural control of antimicrobial handling.

## Data Availability

The original contributions presented in the study are included in the article/Supplementary material, further inquiries can be directed to the corresponding author.
