# Supplementary material for: Syndrome-specific antimicrobial escalation and diagnostic stewardship gaps in large-scale Hungarian pig farms: a cross-sectional survey
Source: Front Vet Sci. 2026 Jul 15;13:1864286. doi: 10.3389/fvets.2026.1864286 (PMC13414140; doi:10.3389/fvets.2026.1864286)
Supplement: Supplementary file 1 [file Table_1.docx]

Supplementary Material

## Supplementary Table S1. Farm-level production-unit coverage and available survey-logistics information

| Farm ID | County | Production units represented in archived workbook | No. of unit-level forms | Survey date range | Respondent framework retained in source materials | Farm-level respondent count retained? |
| --- | --- | --- | --- | --- | --- | --- |
| F01 | Somogy | breeding herd, farrowing unit, nursery unit, grower unit, finisher unit | 5 | 2020-11-09 to 2020-11-13 | Responsible veterinarian; additional manager/owner input where needed | Not retained consistently at farm level |
| F02 | Zala | breeding herd, farrowing unit, nursery unit, finisher unit | 4 | 2020-11-10 to 2020-11-13 | Responsible veterinarian; additional manager/owner input where needed | Not retained consistently at farm level |
| F03 | Somogy | breeding herd, farrowing unit, nursery unit, finisher unit | 4 | 2020-11-16 to 2020-11-19 | Responsible veterinarian; additional manager/owner input where needed | Not retained consistently at farm level |
| F04 | Jász-Nagykun Szolnok | breeding herd, farrowing unit, nursery unit, finisher unit | 4 | 2020-11-26 to 2020-11-29 | Responsible veterinarian; additional manager/owner input where needed | Not retained consistently at farm level |
| F05 | Hajdú-Bihar megye | breeding herd, farrowing unit, nursery unit, finisher unit | 4 | 2020-11-27 to 2020-11-30 | Responsible veterinarian; additional manager/owner input where needed | Not retained consistently at farm level |
| F06 | Bács-Kiskun | breeding herd, farrowing unit, nursery unit, finisher unit | 4 | 2020-12-03 to 2020-12-06 | Responsible veterinarian; additional manager/owner input where needed | Not retained consistently at farm level |
| F07 | Tolna | breeding herd, farrowing unit, nursery unit | 3 | 2020-12-03 to 2020-12-05 | Responsible veterinarian; additional manager/owner input where needed | Not retained consistently at farm level |
| F08 | Szabolcs-Szatmár-Bereg megye | breeding herd, farrowing unit, nursery unit, grower unit, finisher unit | 5 | 2020-12-04 to 2020-12-08 | Responsible veterinarian; additional manager/owner input where needed | Not retained consistently at farm level |
| F09 | Fejér | breeding herd, farrowing unit, nursery unit | 3 | 2020-12-10 to 2020-12-12 | Responsible veterinarian; additional manager/owner input where needed | Not retained consistently at farm level |
| F10 | Baranya | breeding herd, farrowing unit, nursery unit, finisher unit | 4 | 2021-02-17 to 2021-02-20 | Responsible veterinarian; additional manager/owner input where needed | Not retained consistently at farm level |
| F11 | Győr-Moson-Sopron | breeding herd, farrowing unit, nursery unit, grower unit, finisher unit | 5 | 2021-02-20 to 2021-02-24 | Responsible veterinarian; additional manager/owner input where needed | Not retained consistently at farm level |
| F12 | Jász-Nagykun Szolnok | breeding herd, farrowing unit | 2 | 2021-03-01 to 2021-03-02 | Responsible veterinarian; additional manager/owner input where needed | Not retained consistently at farm level |
| F13 | Zala | breeding herd, farrowing unit, nursery unit, finisher unit | 4 | 2021-03-03 to 2021-03-06 | Responsible veterinarian; additional manager/owner input where needed | Not retained consistently at farm level |
| F14 | Komárom-Esztergom | breeding herd, farrowing unit, nursery unit | 3 | 2021-03-04 to 2021-03-06 | Responsible veterinarian; additional manager/owner input where needed | Not retained consistently at farm level |
| F15 | Komárom | finisher unit | 1 | 2021-03-05 | Responsible veterinarian; additional manager/owner input where needed | Not retained consistently at farm level |
| F16 | Komárom-Esztergom | finisher unit | 1 | 2021-03-05 | Responsible veterinarian; additional manager/owner input where needed | Not retained consistently at farm level |

*Farm IDs are anonymized. Production-unit representation was reconstructed from the unit-specific worksheets (farrowing, nursery, grower, finisher, breeding). Individual survey sessions were completed by the responsible veterinarian, with additional operational input from a manager or owner where needed; sessions lasted approximately 30–60 min and were completed individually.*

**Supplementary File S1.** Harmonized English questionnaire used in the study

**Respondent Information and Consent**

I, the undersigned, confirm that I have been informed about the purpose of this questionnaire and about the planned scientific use of the collected information. I understand that the data provided may be used for research and scientific publication in anonymized, aggregated form. By completing this questionnaire, I voluntarily consent to the above-described use and processing of the data.

**Respondent name:** _________________________

**Position / role:** _____________________________

**Institution / company:** ______________________

**Farm name:** ______________________________

**Date of survey:** ____________________________

**Signature:** ________________________________

**Part I. Farm-Level Background Questionnaire**

This section captures the general farm descriptors, herd-size characteristics, production indicators, and herd-health background variables used to characterize the surveyed farms.

**A. Administrative and Structural Descriptors**

1. **Farm name**
2. **County**
3. **Settlement / city**
4. **Country**
5. **Date of survey**
6. **Type of production system**
   - Farrow-to-finish
   - Farrow-to-weaning
   - Farrow-to-nursery
   - Finishing farm
   - Other: __________________
7. **Production units present on the farm** (multiple responses allowed)
   - Farrowing unit
   - Nursery unit
   - Grower (pre-finisher) unit
   - Finisher unit
   - Breeding herd
8. **Genetics present on the farm** (multiple responses allowed)
   - Topigs N
   - DanBred
   - Hypor
   - PIC
   - Other: __________________

**B. Herd-Size Descriptors**

1. **Number of sows**
2. **Number of gilts**
3. **Number of nursery pigs**
4. **Number of grower / pre-finisher pigs**
5. **Number of finisher pigs**
6. **Number of teaser boars**
7. **Number of breeding boars**

**C. Production and Performance Indicators**

1. **Liveborn piglets per sow per year**
2. **Average birth weight (kg)**
3. **Average weaning weight (kg)**
4. **Average weight at the end of the pre-finishing phase (kg)**
5. **Average slaughter weight (kg)**
6. **Number of first-class slaughter pigs sold per year**
7. **Number of out-of-class / culled growing pigs sold per year**
8. **Number of culled sows sold per year**
9. **Number of culled gilts sold per year**
10. **Average lactation length (days)**
11. **Average days in nursery**
12. **Average days in pre-finishing**
13. **Average days in finishing**
14. **Mortality + culling in suckling piglets (%)**
15. **Mortality + culling in nursery pigs (%)**
16. **Mortality + culling in pre-finishers (%)**
17. **Mortality + culling in finishers (%)**
18. **Mortality + culling in sows and gilts (%)**
19. **Average daily gain in suckling piglets**
20. **Average daily gain in nursery pigs**
21. **Average daily gain in pre-finishers**
22. **Average daily gain in finishers**
23. **Feed conversion ratio in suckling piglets**
24. **Feed conversion ratio in nursery pigs**
25. **Feed conversion ratio in pre-finishers**
26. **Feed conversion ratio in finishers**

**D. Carcass Classification / Homogeneity**

1. **Animal homogeneity by production stage**
   Assess separately for lactation, nursery, pre-finisher, and finisher stages:

- 90–100%
- 75–90%
- 50–75%
- <50%

1. **Share of carcasses in S category (S/EUROP)**
2. **Share of carcasses in E category (S/EUROP)**
3. **Share of carcasses in U category (S/EUROP)**
4. **Share of carcasses in R category (S/EUROP)**
5. **Share of carcasses in O category (S/EUROP)**
6. **Share of carcasses in P category (S/EUROP)**

**E. Herd Health Status**

For each variable below, indicate the current status:

- Disease-free
- Under eradication
- Infected
- Not applicable

1. **Aujeszky’s disease**
2. **Leptospirosis**
3. **Brucellosis**
4. **PRRS**
5. **Bordetella bronchiseptica**
6. **Brachyspira hyodysenteriae**
7. **Actinobacillus pleuropneumoniae (APP)**
8. **Mycoplasma hyopneumoniae**

**Part II. Production-Unit-Specific Antimicrobial Stewardship Questionnaire**

This form was completed separately for each relevant production unit. Item numbering **Q1–Q73** is retained here to preserve compatibility with the archived questionnaire logic and with the item references used in the supplementary variable-mapping table. When completing this form, respondents were instructed to base their answers on routine disease occurrence, treatment practice, and stewardship procedures during the **second half of 2020**.

**Section 1. Production-Unit Identifiers (Q1–Q5a)**

**Q1. Farm name**
**Q2. County**
**Q3. Settlement / city**
**Q4. Country**
**Q5. Date of survey**
**Q5a. Production unit category**

- Farrowing unit
- Nursery unit
- Grower (pre-finisher) unit
- Finisher unit
- Breeding herd

**Section 2. Respiratory Syndromes (Q6–Q14)**

**Q6. How would you rate the burden of respiratory disease (PRDC) in this production unit?**

- Absent
- Low
- Moderate (acceptable)
- High
- Very high

**Q7. Are animals showing respiratory symptoms isolated from the rest of the healthy group?**

- No
- Yes, within the same airspace but physically separated
- Yes, in a separate pen, room, or airspace

**Q8. How are animals with respiratory symptoms typically treated?**

- Only the clinically affected animal is treated
- Threshold-based escalation to group treatment
- Immediate treatment of the whole group

**Q9. If threshold-based escalation is used, at what approximate proportion of clinically affected animals is group treatment initiated?**

- 0–10%
- 11–20%
- 21–30%
- 31–40%
- 41–50%

**Q10. Are animals from the respiratory-disease group sampled for bacterial culture and antimicrobial susceptibility testing?**

- Yes, in every case
- Yes, but not in every case
- No

**Q11. If sampling is performed and the group is treated, what is the sampling source?** (multiple responses allowed)

- Sample only the sick animal
- Sample additional group-mates
- Post-mortem sampling

**Q12. Is a recent laboratory susceptibility result available for respiratory-disease cases?**

- Yes
- No
- If yes, attach result where available

**Q13. Is slaughterhouse monitoring used among the diagnostic methods applied for respiratory disease?**

- Yes
- No

**Q14. If slaughterhouse monitoring is used, briefly indicate the recorded respiratory findings or monitoring result.**

- Free-text response

**Section 3. Gastrointestinal Syndromes (Q15–Q23)**

**Q15. How would you rate the burden of gastrointestinal / enteric disease in this production unit?**

- Absent
- Low
- Moderate (acceptable)
- High
- Very high

**Q16. Are animals showing gastrointestinal symptoms isolated from the rest of the healthy group?**

- No
- Yes, within the same airspace but physically separated
- Yes, in a separate pen, room, or airspace

**Q17. How are animals with gastrointestinal symptoms typically treated?**

- Only the clinically affected animal is treated
- Threshold-based escalation to group treatment
- Immediate treatment of the whole group

**Q18. If threshold-based escalation is used, at what approximate proportion of clinically affected animals is group treatment initiated?**

- 0–10%
- 11–20%
- 21–30%
- 31–40%
- 41–50%

**Q19. Are animals from the gastrointestinal-disease group sampled for bacterial culture and antimicrobial susceptibility testing?**

- Yes, in every case
- Yes, but not in every case
- No

**Q20. If sampling is performed and the group is treated, what is the sampling source?** (multiple responses allowed)

- Sample only the sick animal
- Sample additional group-mates
- Post-mortem sampling

**Q21. Is a recent laboratory susceptibility result available for gastrointestinal-disease cases?**

- Yes
- No
- If yes, attach result where available

**Q22. Is slaughterhouse monitoring used among the diagnostic methods applied for gastrointestinal disease?**

- Yes
- No

**Q23. If slaughterhouse monitoring is used, briefly indicate the recorded enteric findings or monitoring result.**

- Free-text response

**Section 4. Joint and Neurological Syndromes (Q24–Q32)**

**Q24. How would you rate the burden of joint and/or neurological disease in this production unit?**

- Absent
- Low
- Moderate (acceptable)
- High
- Very high

**Q25. Are animals showing joint and/or neurological symptoms isolated from the rest of the healthy group?**

- No
- Yes, within the same airspace but physically separated
- Yes, in a separate pen, room, or airspace

**Q26. How are animals with joint and/or neurological symptoms typically treated?**

- Only the clinically affected animal is treated
- Threshold-based escalation to group treatment
- Immediate treatment of the whole group

**Q27. If threshold-based escalation is used, at what approximate proportion of clinically affected animals is group treatment initiated?**

- 0–10%
- 11–20%
- 21–30%
- 31–40%
- 41–50%

**Q28. Are animals from the joint/neurological-disease group sampled for bacterial culture and antimicrobial susceptibility testing?**

- Yes, in every case
- Yes, but not in every case
- No

**Q29. If sampling is performed and the group is treated, what is the sampling source?** (multiple responses allowed)

- Sample only the sick animal
- Sample additional group-mates
- Post-mortem sampling

**Q30. Is a recent laboratory susceptibility result available for joint/neurological-disease cases?**

- Yes
- No
- If yes, attach result where available

**Q31. Is monitoring used among the diagnostic methods applied for joint and/or neurological disease?**

- Yes
- No

**Q32. If monitoring is used, briefly indicate the recorded findings or monitoring result.**

- Free-text response

**Section 5. Urogenital / Reproductive Syndromes (Q33–Q41)**

**Q33. How would you rate the burden of urogenital and/or reproductive disease in this production unit?**

- Absent
- Low
- Moderate (acceptable)
- High
- Very high

**Q34. Are animals showing urogenital / reproductive symptoms isolated from the rest of the healthy group?**

- No
- Yes, within the same airspace but physically separated
- Yes, in a separate pen, room, or airspace

**Q35. How are animals with urogenital / reproductive symptoms typically treated?**

- Only the clinically affected animal is treated
- Threshold-based escalation to group treatment
- Immediate treatment of the whole group

**Q36. If threshold-based escalation is used, at what approximate proportion of clinically affected animals is group treatment initiated?**

- 0–10%
- 11–20%
- 21–30%
- 31–40%
- 41–50%

**Q37. Are animals from the urogenital/reproductive-disease group sampled for bacterial culture and antimicrobial susceptibility testing?**

- Yes, in every case
- Yes, but not in every case
- No

**Q38. If sampling is performed and the group is treated, what is the sampling source?** (multiple responses allowed)

- Sample only the sick animal
- Sample additional group-mates
- Post-mortem sampling

**Q39. Is a recent laboratory susceptibility result available for urogenital/reproductive-disease cases?**

- Yes
- No
- If yes, attach result where available

**Q40. Is monitoring used among the diagnostic methods applied for urogenital / reproductive disease?**

- Yes
- No

**Q41. If monitoring is used, briefly indicate the recorded findings or monitoring result.**

- Free-text response

**Section 6. Antimicrobial Record-Keeping, Storage, and Access (Q42–Q49)**

**Q42. Is there a veterinary medicine / antimicrobial record-keeping system on the farm?**

- Paper-based
- Electronic
- No formal system

**Q43. If records are kept, are medicines documented separately for each production unit or age group?**

- Yes
- No

**Q44. If records are kept, how up to date is the database / record system?**

- 1–5 scale (1 = not at all up to date; 5 = fully up to date)

**Q45. Are antibiotics stored in locked locations?**

- Yes
- No

**Q46. Are the required cooling / temperature conditions for antibiotics consistently respected?**

- 1–5 scale (1 = never; 5 = always)

**Q47. After a product is opened, are the opening date and expiry information indicated on the container?**

- Never
- Sometimes
- Always

**Q48. Are expired antibiotics used despite the fact that their effectiveness is no longer guaranteed?**

- Never
- Sometimes
- Always

**Q49. Who has access to antibiotics on the farm?** (multiple responses allowed)

- Owner
- Farm manager
- Veterinarian
- Shift leader
- Caretaker
- Other: __________________

**Section 7. Injection-Related Administration Practices (Q50–Q59)**

**Q50. Which body site is primarily used for intramuscular administration in pigs?** (multiple responses allowed)

- Neck
- Limb
- Back
- Other: __________________

**Q51. How consistently are animals injected in the same body site according to farm practice?**

- 1–5 scale (1 = never; 5 = always)

**Q52. How much care is taken to ensure that the full dose is actually administered?**

- 1–5 scale (1 = not at all; 5 = completely)

**Q53. If administration is faulty or incomplete, is the animal re-injected?**

- 1–5 scale (1 = never; 5 = always)

**Q54. When combination therapy is used, are products administered separately or drawn up and administered together with one injection?**

- Always separately
- Mixed practice
- Always combined in a single injection

**Q55. After how many animals are syringes changed?**

- After each animal
- After a few animals
- After groups
- Only after marked contamination

**Q56. When a mass-vaccination or dosing device is used, how many products are administered with the same device?**

- Each product has a dedicated device
- More than one product may be used with the same device

**Q57. After how many animals are needles changed?**

- After each animal
- After a few animals
- After groups
- Only when broken

**Q58. Which needle gauge(s) are used?** (multiple responses allowed)

- 22 G
- 21 G
- 20 G
- 19 G
- 18 G
- Other: __________________

**Q59. Which needle length(s) are used?** (multiple responses allowed)

- 10 mm
- 12 mm
- 15 mm
- 20 mm
- 25 mm
- 30 mm
- 40 mm
- 50 mm
- 70 mm
- 80 mm
- 120 mm

**Section 8. Oral Medication and Water-Medication Practices (Q60–Q65)**

**Q60. Which per os administration route(s) are preferred in this production unit?** (multiple responses allowed)

- Individual treatment
- Group treatment in feed
- Group treatment in drinking water

**Q61. If feed medication is used, does the farm purchase ready-made medicated premix or mix it on site?**

- Purchased premix
- Self-mixed

**Q62. Is any drinking-water medication technology available on the farm?**

- Yes
- No

**Q63. Which factors are considered when calculating medication dose or concentration?** (multiple responses allowed)

- Solubility
- Temperature
- Average body weight of the group
- Water intake

**Q64. What type(s) of drinker are used on the farm?** (multiple responses allowed)

- Wet-feeding system
- Nipple drinker
- Cup drinker
- Open-water drinker
- Float drinker

**Q65. When medicines are mixed into drinking water, are the relevant solubility rules observed?**

- 1–5 scale (1 = never; 5 = always)

**Section 9. Stewardship Perceptions and Compliance-Related Items (Q66–Q73)**

**Q66. In the view of farm management, how important is antibiotic use for maintaining animal health on the farm?**

- 1–5 scale (1 = not important; 5 = indispensable)

**Q67. In your opinion, could the currently used antibiotics be replaced by appropriate management while maintaining animal health?**

- 1–5 scale (1 = not at all; 5 = to a very large extent)

**Q68. To what extent do farm workers comply with the rules concerning antibiotic quantity / dose?**

- 1–5 scale (1 = never; 5 = always)

**Q69. To what extent do farm workers comply with the prescribed duration of antibiotic treatment?**

- 1–5 scale (1 = never; 5 = always)

**Q70. To what extent is the prescribed antibiotic protocol followed on the farm?**

- 1–5 scale (1 = never; 5 = always)

**Q71. To what extent are the farm rules for antibiotic storage followed?**

- 1–5 scale (1 = never; 5 = always)

**Q72. How satisfied are you with the effectiveness of antibiotic treatments on the farm?**

- 1–5 scale (1 = very dissatisfied; 5 = very satisfied)

**Q73. What would you change to make antibiotic use more effective on the farm?**

- Free-text response

## Supplementary Table S2. Questionnaire domains, variable mapping, and operational definitions used in the manuscript

| Domain | Variable / construct | Questionnaire item(s) | Original response frame | Derived coding used in manuscript | Applicability denominator | Operational definition / note |
| --- | --- | --- | --- | --- | --- | --- |
| Derived analytical unit | Production unit observation | Farm descriptor + production-stage identifier | farrowing / nursery / finisher / breeding herd (grower unit present only in 3 farms) | Unit-level analytical record | 56 production-unit observations | Main analytical unit used throughout the manuscript; separate grower units were not analysed as a standalone category in the main text. |
| Derived analytical denominator | Affected unit | Any syndrome-specific burden item | absent / low / moderate / high / very high | Affected unit = low, moderate, high, or very high; absent = unaffected | Syndrome-specific | Used as the denominator for isolation, treatment strategy, and culture-and-susceptibility variables. |
| Syndrome burden | Respiratory burden | Q6 | Absent / low / moderate / high / very high | Ordinal burden category retained; non-zero burden collapsed for presence analyses | 56 | Respondent-perceived integrated judgement of routine clinical occurrence, within-unit spread or persistence, clinical severity, and management relevance during the second half of 2020. |
| Syndrome burden | Gastrointestinal burden | Q15 | Absent / low / moderate / high / very high | Ordinal burden category retained; non-zero burden collapsed for presence analyses | 56 | Same operational logic as above. |
| Syndrome burden | Joint/neurological burden | Q24 | Absent / low / moderate / high / very high | Ordinal burden category retained; non-zero burden collapsed for presence analyses | 56 | Same operational logic as above. |
| Syndrome burden | Urogenital burden | Q33 | Absent / low / moderate / high / very high | Ordinal burden category retained; non-zero burden collapsed for presence analyses | 56 | Primarily applicable to breeding herds; other production units were usually recorded as absent or not present. |
| Management response | Isolation implemented | Q7 / Q16 / Q25 / Q34 | No isolation / partitioned within same airspace / separate airspace | Binary derived variable: any partitioned or separate-airspace isolation = Yes; no isolation = No | Affected units only | Used in Figure 3 and Table 3; the raw questionnaire retained isolation subtype. |
| Management response | Treatment strategy | Q8 / Q17 / Q26 / Q35 | Only sick animal treated / threshold-based group treatment / immediate whole-group treatment | Three-category variable retained as reported | Affected units only | Used as the main therapeutic-escalation variable. |
| Management response | Threshold-based escalation trigger | Q9 / Q18 / Q27 / Q36 | Percentage bands of clinical involvement | Reported trigger retained as ordered percentage band; summarised descriptively in Supplementary Table S3 | Threshold-based units only | Applicable only when threshold-based escalation was selected. |
| Diagnostic stewardship | Reported bacterial culture and antimicrobial susceptibility testing | Q10 / Q19 / Q28 / Q37 | No / yes, but not in every case / yes | Binary derived variable: any reported culture+AST activity = Yes; no = No | Affected units only | The questionnaire item referred to culture and susceptibility testing intended to guide treatment decisions. |
| Diagnostic stewardship | Sampling source among units with reported culture+AST | Q11 / Q20 / Q29 / Q38 | Sick animal / whole group / post-mortem sample (multiple responses possible) | Multiple-response descriptive variable | Units with reported culture+AST | Used for narrative interpretation and earlier draft figures; not collapsed into a single exclusive category in the main manuscript. |
| Diagnostic stewardship | Reported resistance test result available | Q12 / Q21 / Q30 / Q39 | Yes / no | Descriptive support variable; not a main table variable in the current manuscript | Units with reported culture+AST | Retained in the archived workbook. |
| Management support | Slaughterhouse monitoring used | Q13 / Q22 / Q31 / Q40 | Yes / no | Descriptive support variable | Production-stage dependent | Not carried into the main manuscript tables. |
| Stewardship indicator | Electronic record-keeping | Q42 | Paper-based / electronic | Binary derived variable: electronic vs paper-based | 56 | Used in Figure 4 and Table 4. |
| Stewardship indicator | Age-group-stratified records | Q43 | Yes / no | Binary variable retained | 56 among units with record system information | Used in Figure 4 and Table 4. |
| Stewardship indicator | Record up-to-dateness | Q44 | Ordinal frequency/completeness response | Descriptive support variable | 56 | Discussed in earlier drafts; not retained as a main summary variable in the present manuscript. |
| Stewardship indicator | Antibiotics stored in locked locations | Q45 | Yes / no | Binary variable retained | 56 | Used in Figure 4 and Table 4. |
| Stewardship indicator | Cold-chain compliance | Q46 | Yes / no / partly | Descriptive support variable | 56 | Archived but not summarised in the current main tables. |
| Stewardship indicator | Post-opening expiry labeling | Q47 | Never / sometimes / always | Derived as no labeling / partial labeling / consistent labeling; the main manuscript emphasises 'no post-opening expiry labeling' and 'consistent labeling' | 56 | Used in Figure 4 and Table 4. |
| Stewardship indicator | Reuse of expired antibiotics | Q48 | Never / sometimes / always | Derived as any reuse vs no reuse | 56 | Used in Figure 4 and Table 4. |
| Stewardship indicator | Personnel with access to antibiotics | Q49 | Owner / farm manager / veterinarian / shift leader / caretaker (multiple responses) | Multiple-response descriptive variable | 56 | Archived but not a main-table variable in the current manuscript. |
| Administration practice | Injection-site practice | Q50–Q54 | Multiple or ordinal response options | Descriptive support variables | 56 or available responses | Archived but not highlighted in the current main tables. |
| Administration practice | Syringe-handling frequency | Q55 | After a few animals / per group / after marked contamination | Categorical variable; the main manuscript highlights 'changed only after marked contamination' | 45 available responses | Used in Figure 4 and Table 4. |
| Administration practice | Number of drugs per dosing device | Q56 | One / more than one | Descriptive support variable | Available responses | Archived but not highlighted in the current main tables. |
| Administration practice | Needle replacement frequency | Q57 | Per individual / per group / only when broken / other | Categorical variable; the main manuscript highlights 'needles changed only when broken' | 56 | Used in Figure 4 and Table 4. |
| Administration practice | Needle gauge | Q58 | Multiple options | Multiple-response descriptive variable | Available responses | Archived but not a main-table variable in the current manuscript. |
| Administration practice | Needle length | Q59 | Multiple options | Multiple-response descriptive variable | Available responses | Archived but not a main-table variable in the current manuscript. |
| Administration practice | Preferred per os route | Q60 | Individual / group feed / group drinking water | Multiple-response descriptive variable | Available responses | Archived; informs interpretation of oral medication infrastructure. |
| Administration practice | Premix sourcing | Q61 | Purchased premix / self-mixed | Categorical variable | Units using feed medication | Archived but not a main-table variable in the current manuscript. |
| Administration practice | Water-medication technology available | Q62 | Yes / no | Binary variable retained | 56 | Used in Figure 4 and Table 4. |
| Administration practice | Dose-calculation factors considered | Q63 | Solubility / temperature / body weight / water intake (multiple responses) | Multiple-response descriptive variable | Units with water-medication technology | Archived and partially discussed in earlier drafts. |

**Supplementary Table S3.** Full distribution of threshold-based treatment triggers by syndrome group and production stage. Values in trigger columns are shown as n/N (%), where N is the number of production units within that syndrome-stage combination that used threshold-based escalation to group treatment. Affected-unit counts are provided for context. “NA” indicates that no unit in that syndrome-stage combination used threshold-based escalation.

| Disease group | Production stage | Affected units (*n*) | Threshold-based escalation (*n*) | 0-10% trigger | 11-20% trigger | 21-30% trigger | 31-40% trigger | 41-50% trigger |
| --- | --- | --- | --- | --- | --- | --- | --- | --- |
| Respiratory | Farrowing | 7 | 2 | 0/2 (0.0%) | 2/2 (100.0%) | 0/2 (0.0%) | 0/2 (0.0%) | 0/2 (0.0%) |
| Respiratory | Nursery | 15 | 9 | 0/9 (0.0%) | 6/9 (66.7%) | 2/9 (22.2%) | 0/9 (0.0%) | 0/9 (0.0%) |
| Respiratory | Finisher | 11 | 7 | 0/7 (0.0%) | 3/7 (42.9%) | 2/7 (28.6%) | 0/7 (0.0%) | 1/7 (14.3%) |
| Respiratory | Breeding herd | 7 | 5 | 0/5 (0.0%) | 3/5 (60.0%) | 1/5 (20.0%) | 0/5 (0.0%) | 0/5 (0.0%) |
| Respiratory | **All stages** | **40** | **23** | **0/23 (0.0%)** | **14/23 (60.9%)** | **5/23 (21.7%)** | **0/23 (0.0%)** | **1/23 (4.3%)** |
| Gastrointestinal | Farrowing | 14 | 4 | 2/4 (50.0%) | 0/4 (0.0%) | 1/4 (25.0%) | 1/4 (25.0%) | 0/4 (0.0%) |
| Gastrointestinal | Nursery | 14 | 10 | 1/10 (10.0%) | 4/10 (40.0%) | 4/10 (40.0%) | 1/10 (10.0%) | 0/10 (0.0%) |
| Gastrointestinal | Finisher | 9 | 6 | 0/6 (0.0%) | 3/6 (50.0%) | 3/6 (50.0%) | 0/6 (0.0%) | 0/6 (0.0%) |
| Gastrointestinal | Breeding herd | 2 | 0 | NA | NA | NA | NA | NA |
| Gastrointestinal | **All stages** | **39** | **20** | **3/20 (15.0%)** | **7/20 (35.0%)** | **8/20 (40.0%)** | **2/20 (10.0%)** | **0/20 (0.0%)** |
| Joint/neurological | Farrowing | 12 | 0 | NA | NA | NA | NA | NA |
| Joint/neurological | Nursery | 16 | 3 | 0/3 (0.0%) | 3/3 (100.0%) | 0/3 (0.0%) | 0/3 (0.0%) | 0/3 (0.0%) |
| Joint/neurological | Finisher | 8 | 1 | 0/1 (0.0%) | 1/1 (100.0%) | 0/1 (0.0%) | 0/1 (0.0%) | 0/1 (0.0%) |
| Joint/neurological | Breeding herd | 7 | 0 | NA | NA | NA | NA | NA |
| Joint/neurological | **All stages** | **43** | **4** | **0/4 (0.0%)** | **4/4 (100.0%)** | **0/4 (0.0%)** | **0/4 (0.0%)** | **0/4 (0.0%)** |
| Urogenital | Farrowing | 1 | 0 | NA | NA | NA | NA | NA |
| Urogenital | Nursery | 0 | 0 | NA | NA | NA | NA | NA |
| Urogenital | Finisher | 0 | 0 | NA | NA | NA | NA | NA |
| Urogenital | Breeding herd | 12 | 0 | NA | NA | NA | NA | NA |
| Urogenital | **All stages** | **13** | **0** | **NA** | **NA** | **NA** | **NA** | **NA** |

**Supplementary Table S4.** Questionnaire-derived variables included in the main analysis, questionnaire item mapping, and analytical denominators by production stage. Counts indicate the number of production-unit observations contributing to each main-analysis variable. For syndrome burden variables, denominators correspond to all production units in the stage. For isolation, treatment strategy, and culture and susceptibility testing, denominators correspond to affected units only. For escalation-threshold variables, denominators correspond to units using threshold-based group treatment only. “Recorded answer” excludes explicit “No data” entries. The main manuscript includes 56 production-unit observations (14 farrowing units, 16 nursery units, 12 finisher units, and 14 breeding herds).

| Domain | Questionnaire item | Main-analysis variable | Farrowing | Nursery | Finisher | Breeding herd | Total | Denominator basis |
| --- | --- | --- | --- | --- | --- | --- | --- | --- |
| Respiratory | 6 | Respiratory syndrome burden score | 14 | 16 | 12 | 14 | 56 | All production units in stage |
| Respiratory | 7 | Respiratory isolation | 7 | 15 | 11 | 7 | 40 | Affected units only |
| Respiratory | 8 | Respiratory treatment strategy | 7 | 15 | 11 | 7 | 40 | Affected units only |
| Respiratory | 9 | Respiratory escalation threshold | 2 | 9 | 7 | 5 | 23 | Threshold-based group-treatment units only |
| Respiratory | 10 | Respiratory culture and susceptibility testing | 7 | 15 | 11 | 7 | 40 | Affected units only |
| Gastrointestinal | 15 | Gastrointestinal syndrome burden score | 14 | 16 | 12 | 14 | 56 | All production units in stage |
| Gastrointestinal | 16 | Gastrointestinal isolation | 14 | 14 | 9 | 2 | 39 | Affected units only |
| Gastrointestinal | 17 | Gastrointestinal treatment strategy | 14 | 14 | 9 | 2 | 39 | Affected units only |
| Gastrointestinal | 18 | Gastrointestinal escalation threshold | 4 | 10 | 6 | 0 | 20 | Threshold-based group-treatment units only |
| Gastrointestinal | 19 | Gastrointestinal culture and susceptibility testing | 14 | 14 | 9 | 2 | 39 | Affected units only |
| Joint/neurological | 24 | Joint/neurological syndrome burden score | 14 | 16 | 12 | 14 | 56 | All production units in stage |
| Joint/neurological | 25 | Joint/neurological isolation | 12 | 16 | 8 | 7 | 43 | Affected units only |
| Joint/neurological | 26 | Joint/neurological treatment strategy | 12 | 16 | 8 | 7 | 43 | Affected units only |
| Joint/neurological | 27 | Joint/neurological escalation threshold | 0 | 3 | 1 | 0 | 4 | Threshold-based group-treatment units only |
| Joint/neurological | 28 | Joint/neurological culture and susceptibility testing | 12 | 16 | 8 | 7 | 43 | Affected units only |
| Urogenital | 33 | Urogenital syndrome burden score | 14 | 16 | 12 | 14 | 56 | All production units in stage |
| Urogenital | 34 | Urogenital isolation | 1 | 0 | 0 | 12 | 13 | Affected units only |
| Urogenital | 35 | Urogenital treatment strategy | 1 | 0 | 0 | 12 | 13 | Affected units only |
| Urogenital | 36 | Urogenital escalation threshold | 0 | 0 | 0 | 0 | 0 | Threshold-based group-treatment units only |
| Urogenital | 37 | Urogenital culture and susceptibility testing | 1 | 0 | 0 | 12 | 13 | Affected units only |
| Stewardship infrastructure | 42 | Electronic record-keeping format | 14 | 16 | 12 | 14 | 56 | All units with recorded answer |
| Stewardship infrastructure | 43 | Age-group-stratified records | 14 | 16 | 12 | 14 | 56 | All units with recorded answer |
| Stewardship infrastructure | 45 | Antibiotics stored in locked locations | 14 | 16 | 12 | 14 | 56 | All units with recorded answer |
| Stewardship infrastructure | 47 | Post-opening expiry labeling | 14 | 16 | 12 | 14 | 56 | All units with recorded answer |
| Stewardship infrastructure | 48 | Reuse of expired antibiotics | 14 | 16 | 12 | 14 | 56 | All units with recorded answer |
| Stewardship infrastructure | 61 | Water-medication technology available | 14 | 16 | 12 | 14 | 56 | All units with recorded answer |
| Injection practice | 55 | Syringe replacement/disinfection frequency | 11 | 13 | 10 | 11 | 45 | All units with recorded answer |
| Injection practice | 57 | Needle replacement frequency | 14 | 16 | 12 | 14 | 56 | All units with recorded answer |

## Supplementary Table S5. Variable-level summary of available and missing observations for questionnaire-derived variables used in the manuscript

| Variable | Expected denominator | Available denominator | Missing n | Reason / applicability rule | Reported in manuscript |
| --- | --- | --- | --- | --- | --- |
| Respiratory burden | 56 | 56 | 0 | All production-unit observations | Figure 1; Figure 2; Table 2 |
| Gastrointestinal burden | 56 | 56 | 0 | All production-unit observations | Figure 1; Figure 2; Table 2 |
| Joint/neurological burden | 56 | 56 | 0 | All production-unit observations | Figure 1; Figure 2; Table 2 |
| Urogenital burden | 56 | 56 | 0 | All production-unit observations | Figure 1; Figure 2; Table 2 |
| Respiratory isolation implemented | 40 | 40 | 0 | Affected respiratory units only | Figure 3; Table 3 |
| Respiratory treatment strategy | 40 | 40 | 0 | Affected respiratory units only | Figure 3; Table 3 |
| Respiratory threshold trigger | 23 | 23 | 0 | Respiratory units with threshold-based escalation only | Supplementary Table S3 |
| Respiratory culture and susceptibility testing | 40 | 40 | 0 | Affected respiratory units only | Figure 3; Table 3 |
| Gastrointestinal isolation implemented | 39 | 39 | 0 | Affected gastrointestinal units only | Figure 3; Table 3 |
| Gastrointestinal treatment strategy | 39 | 39 | 0 | Affected gastrointestinal units only | Figure 3; Table 3 |
| Gastrointestinal threshold trigger | 20 | 20 | 0 | Gastrointestinal units with threshold-based escalation only | Supplementary Table S3 |
| Gastrointestinal culture and susceptibility testing | 39 | 39 | 0 | Affected gastrointestinal units only | Figure 3; Table 3 |
| Joint/neurological isolation implemented | 43 | 43 | 0 | Affected joint/neurological units only | Figure 3; Table 3 |
| Joint/neurological treatment strategy | 43 | 43 | 0 | Affected joint/neurological units only | Figure 3; Table 3 |
| Joint/neurological threshold trigger | 4 | 4 | 0 | Joint/neurological units with threshold-based escalation only | Supplementary Table S3 |
| Joint/neurological culture and susceptibility testing | 43 | 43 | 0 | Affected joint/neurological units only | Figure 3; Table 3 |
| Urogenital isolation implemented | 13 | 13 | 0 | Affected urogenital units only | Figure 3; Table 3 |
| Urogenital treatment strategy | 13 | 13 | 0 | Affected urogenital units only | Figure 3; Table 3 |
| Urogenital threshold trigger | 0 | 0 | 0 | No urogenital units used threshold-based escalation | Supplementary Table S3 |
| Urogenital culture and susceptibility testing | 13 | 13 | 0 | Affected urogenital units only | Figure 3; Table 3 |
| Electronic record-keeping | 56 | 56 | 0 | All production-unit observations | Figure 4; Table 4 |
| Age-group-stratified records | 56 | 56 | 0 | All production-unit observations | Figure 4; Table 4 |
| Antibiotics stored in locked locations | 56 | 56 | 0 | All production-unit observations | Figure 4; Table 4 |
| Post-opening expiry labeling | 56 | 56 | 0 | All production-unit observations | Figure 4; Table 4 |
| Reuse of expired antibiotics | 56 | 56 | 0 | All production-unit observations | Figure 4; Table 4 |
| Water-medication technology available | 56 | 56 | 0 | All production-unit observations | Figure 4; Table 4 |
| Syringe-handling frequency | 56 | 45 | 11 | Complete-case summarization; item-level non-response present | Figure 4; Table 4 |
| Needle replacement frequency | 56 | 56 | 0 | All production-unit observations | Table 4 |

*Missingness was handled by complete-case summarization at the variable level. Most main-manuscript variables were complete after applying the relevant conditional denominator. The syringe-handling item was the main variable*

**Supplementary Table S6.** Exact comparisons of syndrome-specific management responses among affected production units. Percentages were calculated using syndrome-specific affected-unit denominators. Exact 95% confidence intervals were calculated using the Clopper–Pearson method. Omnibus p-values were obtained using Fisher’s exact test across syndrome classes for each outcome.

| Outcome | Syndrome class | Positive/ affected units | % | Exact 95% CI | Omnibus p-value |
| --- | --- | --- | --- | --- | --- |
| Group treatment | Respiratory | 27/40 | 67.5 | 50.9–81.4 | 0.0001 |
|  | Gastrointestinal | 30/39 | 76.9 | 60.7–88.9 |  |
|  | Joint/neurological | 4/43 | 9.3 | 2.6–22.1 |  |
|  | Urogenital | 0/13 | 0.0 | 0.0–24.7 |  |
| Isolation | Respiratory | 14/40 | 35.0 | 20.6–51.7 | 0.0313 |
|  | Gastrointestinal | 22/39 | 56.4 | 39.6–72.2 |  |
|  | Joint/neurological | 27/43 | 62.8 | 46.7–77.0 |  |
|  | Urogenital | 4/13 | 30.8 | 9.1–61.4 |  |
| Resistance-oriented sampling | Respiratory | 24/40 | 60.0 | 43.3–75.1 | 0.7816 |
|  | Gastrointestinal | 24/39 | 61.5 | 44.6–76.6 |  |
|  | Joint/neurological | 24/43 | 55.8 | 39.9–70.9 |  |
|  | Urogenital | 6/13 | 46.2 | 19.2–74.9 |  |

Abbreviation: CI, confidence interval.

**Supplementary File S2.** STROBE checklist

| Item no. | Checklist item | Recommendation | Location in manuscript |
| --- | --- | --- | --- |
| 1 | Title and abstract | Indicate the study design with a commonly used term in the title or the abstract; provide an informative and balanced summary of what was done and what was found. | Title; Abstract |
| 2 | Background/rationale | Explain the scientific background and rationale for the investigation being reported. | Introduction, paragraphs 1–6 |
| 3 | Objectives | State specific objectives, including any prespecified hypotheses. | End of Introduction |
| 4 | Study design | Present key elements of study design early in the paper. | Materials and methods / Study design and farm recruitment |
| 5 | Setting | Describe the setting, locations, and relevant dates, including periods of recruitment and data collection. | Materials and methods / Study design and farm recruitment |
| 6a | Participants | Give the eligibility criteria, and the sources and methods of selection of participants. | Materials and methods / Study design and farm recruitment |
| 6b | Participants | For matched studies, give matching criteria and number of exposed and unexposed (if applicable). | Not applicable |
| 7 | Variables | Clearly define all outcomes, exposures, predictors, potential confounders, and effect modifiers. Give diagnostic criteria, if applicable. | Materials and methods / Variables of interest; Supplementary Tables S1–S6 |
| 8 | Data sources/measurement | For each variable of interest, give sources of data and details of methods of assessment; describe comparability of assessment methods if there is more than one group. | Materials and methods / Study design and farm recruitment; Questionnaire structure and analytical unit; Variables of interest |
| 9 | Bias | Describe any efforts to address potential sources of bias. | Materials and methods / Study design and farm recruitment; Discussion / Limitations |
| 10 | Study size | Explain how the study size was arrived at. | Materials and methods / Study design and farm recruitment; respondent-reported survey sample described |
| 11 | Quantitative variables | Explain how quantitative variables were handled in the analyses; describe groupings if applicable. | Materials and methods / Variables of interest; Statistical analysis |
| 12a | Statistical methods | Describe all statistical methods, including those used to control for confounding. | Materials and methods / Statistical analysis |
| 12b | Statistical methods | Describe any methods used to examine subgroups and interactions. | Materials and methods / Statistical analysis; syndrome-specific comparisons |
| 12c | Statistical methods | Explain how missing data were addressed. | Materials and methods / Statistical analysis; Supplementary Table S5 |
| 12d | Statistical methods | If applicable, explain how loss to follow-up was addressed. | Not applicable (cross-sectional survey) |
| 12e | Statistical methods | Describe any sensitivity analyses. | Not performed; clustering limitation acknowledged in Statistical analysis and Discussion |
| 13a | Participants | Report numbers of individuals at each stage of study (eligible, included, analysed). | Materials and methods / Study design and farm recruitment; Table 1; Supplementary Table S1 |
| 13b | Participants | Give reasons for non-participation at each stage. | Materials and methods / Study design and farm recruitment |
| 13c | Participants | Consider use of a flow diagram. | Not provided; recruitment numbers described textually |
| 14a | Descriptive data | Give characteristics of study participants and information on exposures and potential confounders. | Results / Farm characteristics; Table 1; Supplementary Table S1 |
| 14b | Descriptive data | Indicate number of participants with missing data for each variable of interest. | Materials and methods / Statistical analysis; Supplementary Table S5 |
| 15 | Outcome data | Report numbers of outcome events or summary measures. | Results; Tables 2–4; Supplementary Tables S3, S4 and S6 |
| 16a | Main results | Give unadjusted estimates and, if applicable, confounder-adjusted estimates and their precision. | Results / inferential paragraphs; Supplementary Table S6 |
| 16b | Main results | Report category boundaries when continuous variables were categorized. | Materials and methods / Variables of interest; Supplementary Table S2 |
| 16c | Main results | If relevant, consider translating estimates of relative risk into absolute risk. | Not applicable |
| 17 | Other analyses | Report other analyses done, e.g., subgroup analyses, interactions, sensitivity analyses. | Results / syndrome-specific comparisons; Supplementary Tables S3 and S6 |
| 18 | Key results | Summarise key results with reference to study objectives. | Discussion, opening paragraphs; Conclusion |
| 19 | Limitations | Discuss limitations of the study, taking into account sources of potential bias or imprecision. | Discussion / Limitations |
| 20 | Interpretation | Give a cautious overall interpretation of results considering objectives, limitations, multiplicity of analyses, and evidence from similar studies. | Discussion |
| 21 | Generalisability | Discuss the generalisability of the study results. | Discussion / Limitations and strengths |
| 22 | Funding | Give the source of funding and the role of the funders. | Funding statement |
